# Supplementary material for: Assessing the Quality of ChatGPT’s Dietary Advice for College Students from Dietitians’ Perspectives
Source: Nutrients. 2024 Jun 19;16(12):1939. doi: 10.3390/nu16121939 (PMC11206595; doi:10.3390/nu16121939)
Supplement: Supplementary file 1 [file nutrients-16-01939-s001.zip › nutrients-3052113-supplementary.pdf]

Supplement

Scenarios, Corresponding Indicators and Prompts for ChatGPT

Scenario/Prompt	Indicators [23]
**S1: Balanced Eating**<br>College student Casey's busy academic and club activities often leave him eating out and consuming an unbalanced diet, primarily consisting of bread, microwaveable food, or sugary drinks, with late-night snacks at night markets. Recently, he has been experiencing constipation and acne on his face, leading him to suspect that his diet and lifestyle may be the problem. He is now eager to learn how to adjust them to maintain his physical health.<br><br>*[Prompt] Due to an unbalanced diet, I am experiencing irregular bowel movements and acne breakouts. How should I adjust my diet?*	1-1-1 Understand meaning of the Daily Food Guide<br>1-1-2 Understand classification of the 6 food groups and dietary values<br>1-1-3 Understand influence of well-balanced diets on health<br>1-5-1 Understand influence of adequate intake of wholegrain foods on health<br>2-1-1 Analyze types of foods from 6 groups and recommended quantity of an individual's daily diet<br>2-5-1 Analyze the types and quantity of wholegrain foods an individual should consume daily<br>3-1-1 Appraise whether an individual's daily diet conforms to the recommended types and quantity of foods from the 6 groups<br>4-1-1 Choose or modify personal diet based on the recommended types and quantity of foods from 6 groups
**S2: Fit Weight**<br>College student Xuan discovered that he belongs to the obese population during a health check-up. In order to lose weight, he started to use intermittent fasting and weight loss products that were introduced on the internet. However, he has recently experienced symptoms such as palpitations, chest tightness, dizziness, and profuse sweating, which have made him feel very anxious. Therefore, he is eager to know how to improve his current condition.<br><br>*[Prompt] How can I achieve healthy weight loss while avoiding side effects like palpitations, chest tightness, dizziness, and excessive sweating, commonly associated with fasting or weight loss products?*	1-2-1 Understand relationship between healthy body weight and risk of chronic diseases<br>1-2-2 Understand definition of healthy body weight and calculation of body mass index<br>1-3-1 Understand purpose of physical activity<br>2-2-1 Analyze personal healthy body weight and energy requirements<br>3-2-1 Appraise whether an individual's daily diet conforms to personal energy requirements<br>3-3-1 Appraise whether an individual's physical activity is appropriate and adequate<br>4-2-1 Choose or modify personal diet based on personal energy requirements<br>4-5-1 Choose the low oil cooking method and dishes for personal health reasons

**S3: Dining Out Well**<br>College student Ming is a fan of convenience store products. However, one day she saw a news report indicating that frequent consumption of these foods can result in excessive sodium intake and accidental consumption of high calories, leading to increased risk of stroke, heart disease, and kidney disease. Ming started to worry about her health and wants to reduce her intake of high-sodium and high-calorie foods, but she doesn't know how to get started.<br><br>*[Prompt] When dining at convenience stores, how can I make healthy choices to reduce the intake of high-calorie and high-sodium foods?*	1-11-1 Understand food hygiene and safety precautions when buying foods (such as reading food labels and source of production)<br>【Analyze】<br>2-2-2 Analyze the energy of packaged foods based on nutrition facts<br>2-6-1 Analyze the sodium content of packaged foods based on nutrition facts<br>2-6-2 Analyze the types of high sodium foods eaten in daily life<br>2-6-3 Analyze the types of high calorie low nutrient density foods
**S4: Fewer Processed Foods**<br>College student Ali and his friends in the department organized a hot pot party and noticed that there were a lot of meat slices and processed hot pot ingredients, but very few vegetables. Ali has recently started to pay attention to a balanced diet and feels that this type of diet may not be very healthy. He wants to add some healthier ingredients to the hot pot but doesn't know how to make it healthier.<br><br>*[Prompt] What are some ingredients and vegetables I can choose to make my hot pot experience healthier?*	3-8-1 Appraise whether an individual's diet contains too many excessively processed foods<br>4-8-1 Choose not excessively processed foods for personal health reasons<br>4-8-2 Choose plant foods more often for personal health reasons
**S5: Limit Sugary Drinks**<br>College student Allen enjoys drinking bubble tea, but recently he has noticed an increase in his weight and spending, making him reconsider the frequency of his bubble	1-7-1 Understand that drinking enough boiled water is important to health<br>1-7-2 Understand influence of drinking too many sugar-sweetened beverages on health

tea consumption. However, with the hot weather, he is unsure of how to quench his thirst without turning to bubble tea.<br><br>*[Prompt] When thirsty during hot weather but reluctant to drink sugary beverages for fear of gaining weight, what healthy thirst-quenching methods can you recommend?*	2-7-1 Analyze the calories of commercial sugar-sweetened beverages<br>3-7-1 Appraise whether an individual's daily boiled water intake is sufficient

Note. (a) The code of NL abilities (e.g., 1-1-1): the first number refers to NL type: 1-understand, 2-analyze, 3-appraise, and 4-apply; the middle number refers to different health themes according to the Dietary Guidelines for Taiwanese; and the third number corresponds to the serial number.
